# The influence of endometrial thickness on clinical pregnancy outcomes in early-follicular long-acting and midluteal short-acting GnRH agonist long protocols: a large retrospective study

**DOI:** 10.3389/fendo.2025.1637587

**Published:** 2025-11-26

**Authors:** Chen Wang, Congying Yang, Wei Dai, Liping Han, Huijuan Kong, Guidong Yao, Xiao Wang, Zhiqin Bu, Yuhe Peng, Jun Zhai

**Affiliations:** 1 Center for Reproductive Medicine, The First Affiliated Hospital of Zhengzhou University, Zhengzhou, Henan, China; 2 Henan Key Laboratory of Reproduction and Genetics, The First Affiliated Hospital of Zhengzhou University, Zhengzhou, China; 3 Henan Provincial Obstetrical and Gynecological Diseases, Reproductive Medicine, Clinical Research Center, The First Affiliated Hospital of Zhengzhou University, Zhengzhou, Henan, China

**Keywords:** endometrial thickness, early-follicular long-acting GnRH agonist protocol, midluteal short-acting GnRH agonist long protocol, endometrial receptivity, restricted cubic spline (RCS)

## Abstract

**Objective:**

To evaluate the impact of endometrial thickness (EMT) variations on clinical outcomes in two distinct ovarian stimulation protocols: the early-follicular long-acting GnRH agonist protocol and the midluteal short-acting GnRH agonist long protocol.

**Methods:**

This retrospective cohort study analyzed 21,290 first-time IVF/ICSI fresh embryo transfer cycles conducted at the Reproductive Center of the First Affiliated Hospital of Zhengzhou University between January 2013 and December 2020. Restricted cubic spline (RCS) analysis was employed to assess the relationship between EMT and clinical pregnancy outcomes.

**Results:**

In the early-follicular long-acting GnRH agonist protocol group, both clinical pregnancy and live birth rates increased with EMT up to 10.6 mm, beyond which the rates plateaued. Conversely, in the midluteal short-acting GnRH agonist long protocol group, a continuous positive correlation was observed between EMT and both clinical pregnancy and live birth rates. Overall, the early-follicular long-acting protocol demonstrated superior pregnancy outcomes compared to the midluteal short-acting protocol when EMT was less than 15 mm. However, when EMT was ≥15 mm, both protocols yielded comparable clinical pregnancy and live birth rates.

**Conclusion:**

The study indicates that in the early-follicular long-acting GnRH agonist protocol, increasing EMT up to 10.6 mm is associated with improved clinical pregnancy and live birth rates, with no further benefits observed beyond this threshold. In contrast, the midluteal short-acting GnRH agonist long protocol exhibits a continuous positive relationship between EMT and pregnancy outcomes. Overall, the early-follicular long-acting protocol offers better clinical outcomes for patients with EMT less than 15 mm, while both protocols perform similarly when EMT is ≥15 mm.

## 1 Background

Successful pregnancy depends on both high-quality embryos and optimal endometrial receptivity. Research indicates that approximately two-thirds of implantation failures are attributed to inadequate endometrial receptivity and defective embryo–endometrium interactions (1). Endometrial thickness on the day of human chorionic gonadotropin (hCG) administration has been studied as a potential prognostic factor for assisted reproductive technology (ART) outcomes and is considered one of the markers of endometrial receptivity (1–4). Multiple studies have reported that thinner endometrial thickness is associated with lower clinical pregnancy rates, higher miscarriage rates, and increased risks of small-for-gestational-age (SGA) infants. Conversely, thicker endometrial thickness (>7–8 mm) is correlated with improved outcomes in *in vitro* fertilization (IVF) (5–10). Gonadotropin-releasing hormone (GnRH) agonists have been utilized for years to control ovarian stimulation, resulting in lower spontaneous ovulation rates and higher pregnancy rates. There are two primary protocols: the early-follicular long-acting GnRH agonist protocol (EFLL) and the midluteal short-acting GnRH agonist long protocol (MLSL). Studies have shown that the EFLL protocol yields higher clinical pregnancy and live birth rates compared to the MLSL protocol (11, 12). However, it remains unclear whether this advantage persists as endometrial thickness increases on the day of human chorionic gonadotropin (hCG) administration, and whether the trends in the relationship between endometrial thickness and pregnancy outcomes differ between these two protocols.

This retrospective analysis utilized clinical data from 21,290 patients who underwent their first IVF/ICSI fresh embryo transfer cycles at the Reproductive Center of the First Affiliated Hospital of Zhengzhou University between January 2013 and December 2020. The study employed Restricted Cubic Spline (RCS) analysis to examine the dynamic relationship between endometrial thickness on the day of hCG administration and pregnancy outcomes under two ovarian stimulation protocols: the Early-Follicular Long-Acting GnRH Agonist Protocol (EFLL) and the Midluteal Short-Acting GnRH Agonist Long Protocol (MLSL). The analysis aimed to identify the optimal endometrial thickness thresholds for achieving the best pregnancy outcomes and to assess potential interaction effects between endometrial thickness and stimulation protocols. This large-sample retrospective study provides valuable insights into the dynamic association between endometrial thickness and pregnancy outcomes under different stimulation protocols, offering clinical guidance for protocol selection.

## 2 Materials and methods

### 2.1 Study design

A retrospective analysis was conducted on the clinical data of 21,290 patients who underwent their first IVF/ICSI fresh embryo transfer cycles at the Reproductive Center of the First Affiliated Hospital of Zhengzhou University between January 2013 and December 2020. After propensity score matching, 6,974 patients were assigned to the early-follicular long-acting GnRH agonist protocol (EFLL) group, and 2,324 patients to the midluteal short-acting GnRH agonist long protocol (MLSL) group.

Inclusion Criteria: Patients who underwent their first IVF/ICSI cycle with fresh embryo transfer at the Reproductive Center of the First Affiliated Hospital of Zhengzhou University between January 2013 and December 2020.

Exclusion Criteria: Patients with untreated hydrosalpinx, hyperprolactinemia, diabetes mellitus, or other endocrine disorders; uterine malformations; preimplantation genetic diagnosis or screening; uterine fibroids >3 cm in diameter compressing the endometrium; endometriosis or adenomyosis; intrauterine adhesions; cervical insufficiency; history of tuberculosis; or unoperated endometrial polyps.

Patients were categorized into groups based on their endometrial thickness on their human chorionic gonadotropin day; thicknesses of <7 mm, >16 mm, and 1-mm increments for the remaining measurements were noted, resulting in a total of 11 groups.

This study was approved by the Research and Clinical Trials Ethics Committee of the First Affiliated Hospital of Zhengzhou University (project number 2024-KY-0412-001). In accordance with national legislation, institutional requirements, and the principles of the Declaration of Helsinki, written informed consent was not required for this study.

### 2.2 Controlled superovulation protocols

#### 2.2.1 Early follicular long-acting GnRH agonist protocol

Patients receive a 3.75 mg dose of the long-acting GnRH agonist triptorelin (Pfizer Pharmaceutical Co., Ltd., Germany) on days 2–3 of their menstrual cycle, following natural menstruation or oral dydrogesterone administration. Serum levels of follicle-stimulating hormone (FSH), luteinizing hormone (LH), estradiol (E2), and progesterone (P) are measured 28 days after injection. Follicular development is monitored via transvaginal ultrasound.

#### 2.2.2 Midluteal short-acting GnRH agonist long protocol

For patients with regular menstrual cycles, the protocol begins on days 21–22 of the natural cycle. For those with irregular cycles, oral contraceptive pills containing drospirenone and ethinylestradiol (Bayer Pharmaceuticals, Germany) are administered for 21 days, starting on day 5 of menstruation. From day 16 of oral contraceptive use, subcutaneous injections of short-acting GnRH agonist triptorelin (IPSEN Biotechnology, France) are given at a daily dose of 0.1 mg for 14–16 days. During this period, oral contraceptive pills are continued at a daily dose of 1 tablet. After injection, serum levels of FSH, LH, E2, and P are measured, and follicular development is monitored via transvaginal ultrasound.

When pituitary downregulation criteria are met (FSH < 5 U/L, LH < 3 U/L, E2 < 50 pg/mL, antral follicle diameter approximately 3–5 mm, no ovarian cysts >10 mm), controlled ovarian stimulation (COS) is initiated with gonadotropins (Gn). Gn starting doses are determined based on the patient’s body mass index (BMI). Gn doses are adjusted according to follicular growth and serum E2 levels. When two or more follicles reach 18 mm in diameter, or over two-thirds of follicles reach 16 mm, recombinant human chorionic gonadotropin (hCG, Merck Serono, Italy) is administered to trigger ovulation. Oocyte retrieval is performed 36–37 hours after hCG administration. Luteal phase support was initiated on the day of oocyte retrieval using intramuscular progesterone injections (60 mg/day; Zhejiang Xianju Pharmaceutical Co., Ltd., China) and continued until pregnancy test results were confirmed. This regimen was implemented according to our center’s routine clinical practice and relevant guideline recommendations. *In vitro* fertilization (IVF) or intracytoplasmic sperm injection (ICSI) is used for fertilization.

### 2.3 Research indicators

We measured serum β-human chorionic gonadotropin levels 14 days after embryo transfer. Clinical pregnancy was confirmed by the presence of a gestational sac on ultrasonography, which was performed 35 days after transfer. High-quality embryo rate = number of high-quality embryos/number of 2PN cleavages*100%; biochemical pregnancy rate = number of HCG-positive cycles/number of transfer cycles*100%; implantation rate = number of visible gestational sacs under ultrasound/number of transferred embryos*100%; clinical pregnancy rate = number of clinical pregnancy cycles/total number of transfer cycles*100%; live birth rate = number of live birth cycles/total number of transfer cycles*100%; abortion rate = number of abortion cycles/number of clinical pregnancy cycles*100%; ectopic pregnancy rate = number of ectopic pregnancy cycles/number of clinical pregnancy cycles*100%; premature birth rate = number of premature birth cycles/number of clinical pregnancy cycles*100%.

### 2.4 Statistical analysis

Due to the significantly larger sample size in the early-follicular long-acting GnRH agonist protocol group compared to the midluteal short-acting GnRH agonist long protocol group, propensity score matching (PSM) was employed to ensure comparability between the two groups. A 1:3 matching ratio was applied based on covariates including age, BMI, infertility type, antral follicle count, FSH, LH, E2, testosterone, fertilization method, and number of embryos transferred. The matching process was performed using the nearest neighbor matching algorithm with a caliper width set at 0.02 and without replacement. After matching, the balance between groups was evaluated using the standardized mean difference (SMD), with all covariates showing SMD values <0.1, indicating good matching quality.

In the original cohort, there were 2,361 cases in the midluteal short-acting GnRH agonist long protocol group and 18,929 cases in the early-follicular long-acting GnRH agonist protocol group. After matching, 2,324 cases from the midluteal short-acting GnRH agonist long protocol group and 6,974 cases from the early-follicular long-acting GnRH agonist protocol group were included. The matching ratio did not strictly achieve 1:3, as 37 cases from the midluteal short-acting GnRH agonist long protocol group were excluded due to propensity scores falling outside the caliper range. Among the remaining 2,324 cases, some were matched to 2–4 cases from the early-follicular long-acting GnRH agonist protocol group, resulting in an average matching ratio close to 3:1 (6,974/2,324 ≈ 3.00), with negligible differences.

Continuous variables were assessed for normality using the Shapiro-Wilk test. Since all continuous variables were normally distributed (*P* > 0.05), t-tests were performed for comparisons between groups. Categorical variables were expressed as percentages and compared using chi-square tests. Multivariate logistic regression was conducted to adjust for potential confounders, including age, BMI, infertility type, number of embryos transferred, embryo or blastocyst transfer, initial Gn dosage, total Gn dosage, antral follicle count, number of oocytes retrieved, and stimulation protocol. Adjusted odds ratios (aORs) with 95% confidence intervals (CIs) were calculated to examine the association between endometrial thickness and pregnancy outcomes. Interaction effects between protocol type and endometrial thickness were evaluated by introducing an interaction term (“protocol × endometrial thickness”) into the model and testing its significance using the likelihood ratio test.

Restricted cubic spline (RCS) analysis was used to examine the relationship between endometrial thickness and clinical pregnancy rate, live birth rate, miscarriage rate, and preterm birth rate. All models were adjusted for age, body mass index (BMI), type of infertility, number of transferred embryos, embryo or blastocyst stage at transfer, initial gonadotropin dose, total gonadotropin dose, antral follicle count (AFC), number of retrieved oocytes, and ovarian stimulation protocol.

To balance model complexity and the risk of overfitting, the RCS model was specified with four knots placed at the 5th, 35th, 65th, and 95th percentiles of endometrial thickness, with three degrees of freedom. The likelihood ratio test and Wald test were used to assess the significance of nonlinearity (P for nonlinearity < 0.05 indicating a significant nonlinear relationship). If a nonlinear relationship was detected, piecewise logistic regression models were constructed based on the identified inflection points.

All statistical analyses were performed using SPSS version 25.0 (IBM Corporation, Armonk, NY, USA) and R version 4.2.1. Two-sided P-values < 0.05 were considered statistically significant.

## 3 Results

### 3.1 General characteristics and clinical outcomes of patients undergoing different ovarian stimulation protocols

**Table 1** shows that after propensity score matching, there were no significant differences between the two groups in terms of age, BMI, infertility type, antral follicle count, FSH, LH, E2, T, and embryo transfer type (all *P* > 0.05). Compared to the midluteal short-acting GnRH agonist group, the early-follicular long-acting GnRH agonist group had a higher number of oocytes retrieved, greater endometrial thickness on the hCG day, higher biochemical pregnancy rate, clinical pregnancy rate, and live birth rate, and lower ectopic pregnancy rate (all *P* < 0.05). However, there were no significant differences between the two groups in miscarriage rate and preterm birth rate (all *P* > 0.05). Overall, the midluteal short-acting GnRH agonist protocol was associated with a lower incidence of pregnancy-related complications, specifically a lower rate of gestational diabetes mellitus (*P* < 0.001), while there were no significant differences between the two groups in terms of pregnancy-induced hypertension, premature rupture of membranes, and intrauterine distress (all *P* > 0.05, **Table 1**).

**Table 1:** General characteristics and clinical outcomes of patients undergoing different ovulation induction protocols.

Variables	EFLL (n=6974)	MLSL (n=2324)	*P*
Age (n%)			0.400
<35 years	5229 (75.0)	1722 (74.1)	
≥35 years	1745 (25.0)	602 (25.9)	
BMI (Kg/m2)	23.18 ± 3.24	23.12 ± 3.34	0.770
Type of infertility (n%)			0.880
Primary infertility	3599 (51.6)	1195 (51.4)	
Secondary infertility	3375 (48.4)	1129 (48.6)	
Number of sinus follicles (n)	13.78 ± 7.08	13.69 ± 6.97	0.550
FSH (mIU/ml)	7.12 ± 2.48	7.13 ± 2.28	0.740
LH (mIU/ml)	6.02 ± 5.40	6.22 ± 4.66	0.110
E2 (pg/ml)	53.80 ± 137.54	56.96 ± 142.65	0.340
T (ng/ml)	0.53 ± 3.37	0.57 ± 3.72	0.550
Insemination method (n%)			0.640
IVF	4927 (70.6)	1630 (70.1)	
ICSI	2047 (29.4)	694 (29.9)	
Embryos/blastocysts transferred (n%)			0.570
Embryo	5863 (84.1)	1942 (83.6)	
Blastocysts	1111 (15.9)	382 (16.4)	
Number of embryos transferred (n)	1.74 ± 0.44	1.76 ± 0.43	0.102
Endometrial Thickness on hCG Trigger Day (mm)	12.29 ± 2.55	11.61 ± 2.42	<0.001
Number of Oocytes Retrieved (n)	12.29 ± 2.55	11.61 ± 2.42	<0.001
High-Quality Embryo Rate (%)	64.64 ± 0.28	64.45 ± 0.24	0.76
Embryo implantation rate (%)	48.32 ± 42.31	36.64 ± 41.28	<0.001
Biochemical pregnancy rate (%)	67.8 (4731/6974)	54.0 (1255/2324)	<0.001
Clinical pregnancy rate (%)	63.9 (4454/6974)	50.0 (1162/2324)	<0.001
Live birth rate (%)	54.5 (3800/6974)	41.0 (952/2324)	<0.001
Miscarriage rate (%)	13.0 (579/4454)	14.5 (169/1162)	0.175
Ectopic Pregnancy Rate (%)	1.9 (85/4454)	3.8 (44/1162)	<0.001
Preterm birth rate (%)	12.4 (552/4454)	13.8 (160/1162)	0.210
Gestational Complications (%)	10.2 (4533/4454)	6.7 (78/1162)	<0.001
Gestational Hypertension (%)	2.8 (123/4454)	2.8 (33/1162)	0.885
Gestational Diabetes Mellitus (%)	3.4 (153/4454)	1.2 (14/1162)	<0.001
Premature Rupture of Membranes (%)	3.5 (158/4454)	2.4 (28/1162)	0.054
Intrauterine Fetal Distress (%)	0.4 (19/4454)	0.2 (2/1162)	0.206

Continuous data, mean ± SD; categorical data, n (%), BMI=body mass index; EFLL=early-follicular long-acting GnRH agonist protocol; MLSL= midluteal short-acting GnRH agonist long protocol; ART= assisted reproductive technology.

### 3.2 Clinical outcomes associated with different endometrial thicknesses

**Figure 1:**
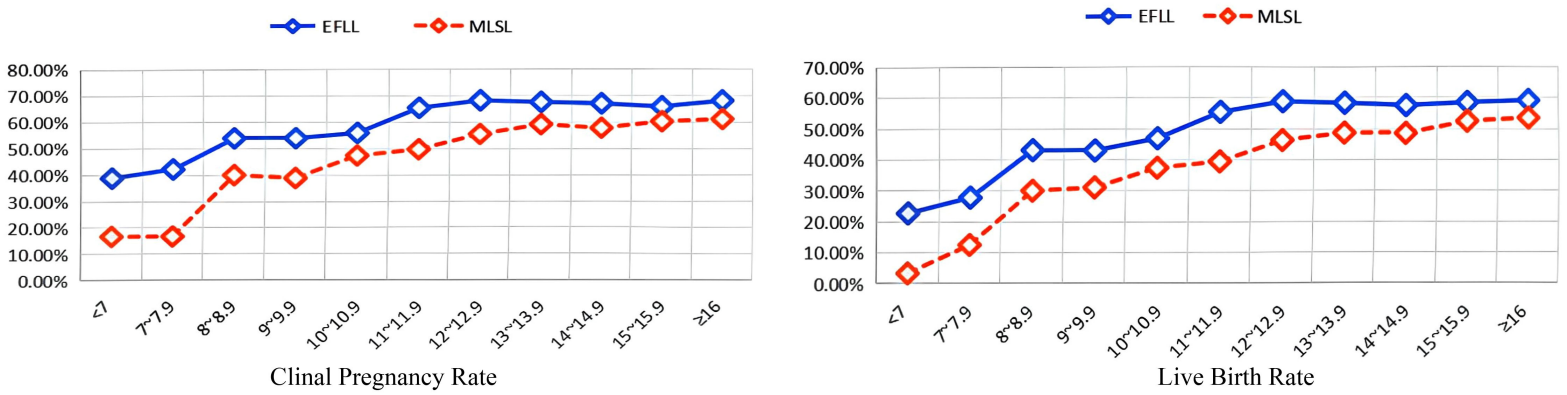
Relationship between endometrial thickness and clinical outcomes in different controlled ovulation induction protocols. EFLL, early-follicular long-acting GnRH agonist protocol; MLSL, midluteal short-acting GnRH agonist long protocol.

As shown in **Table 2** and **Figure 1**, when the endometrial thickness is less than 15 mm, the clinical pregnancy rate and live birth rate in the follicular-phase long protocol are consistently higher than those in the luteal-phase short protocol, with statistical significance (all *P* < 0.05, **Table 2**). However, when the endometrial thickness is 15 mm or greater, there is no significant difference in clinical pregnancy rate and live birth rate between the two groups, both exhibiting similar pregnancy outcomes (all *P* > 0.05, **Table 2**).

**Table 2:** Clinical outcomes of different endometrial thicknesses in different controlled ovulation induction protocols.

Endometrial thickness (mm)	Clinical pregnancy rate (%)		*P*	Live birth rate (%)		*P*
	EFLL	MLSL		EFLL	MLSL	
<7	38.7% (29/75)	16.7% (5/30)	0.03	22.7% (17/75)	3.3% (1/30)	0.018
7~7.9	42.1% (53/126)	16.7% (8/48)	0.002	27.8% (35/126)	12.5% (6/48)	0.034
8~8.9	53.8% (155/288)	40.0% (64/160)	0.005	43.1% (124/288)	30.0% (48/160)	0.006
9~9.9	53.8% (222/413)	38.8% (85/219)	<0.001	42.9% (68/413	31.1% (68/219)	0.004
10~10.9	55.9% (392/701)	47.4% (152/321)	0.011	46.9% (329/701)	37.4% (120/321)	0.004
11~11.9	65.4% (618/945)	49.7% (169/340)	<0.001	55.4% (524/945)	39.4% (134/340)	<0.001
12~12.9	68.2% (853/1250)	55.5% (222/400)	<0.001	58.8% (735/1250)	46.3% (185/400)	<0.001
13~13.9	67.5% (686/1017)	59.2% (186/314)	0.007	58.3% (593/1017)	48.7% (153/314)	0.003
14~14.9	67.0% (592/884)	57.8% (137/237)	0.009	57.4% (507/884)	48.5% (115/237)	0.015
15~15.9	65.8% (400/608)	60.3% (76/126)	0.242	58.4% (355/608)	52.4% (66/126)	0.215
≥16	68.1% (454/667)	61.2% (79/129)	0.131	59.1% (394/667)	53.5% (69/129)	0.24

Continuous data, mean ± SD; categorical data, n (%); EFLL=early-follicular long-acting GnRH agonist protocol; MLSL= midluteal short-acting GnRH agonist long protocol.

### 3.3 Interaction between ovulation induction protocols and endometrial thickness

**Figure 2:**
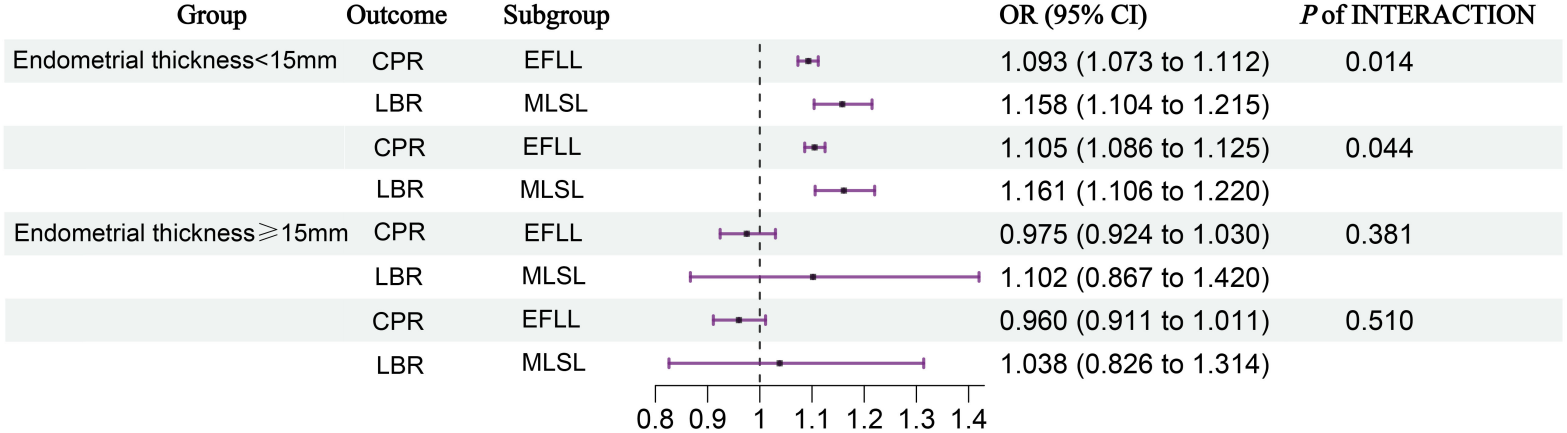
Interaction analysis between ovulation induction protocols and endometrial thickness. We adjusted for age, BMI, infertility type, number of embryos transferred, type of embryos transferred, Gn initiation, total Gn, number of sinus follicles, number of follicle retrievals, and controlled ovulation induction protocol. CI, confidence interval; EFLL, early-follicular long-acting GnRH agonist protocol; MLSL, midluteal short-acting GnRH agonist long protocol; CPR, clinical pregnancy rate; LBR, live birth rate.

As depicted in **Figure 2**, when endometrial thickness is less than 15 mm, a significant interaction exists between the ovulation induction protocol and endometrial thickness (all *P* < 0.05, **Figure 2**). However, when the endometrial thickness is 15 mm or greater, this interaction is no longer observed (*P* > 0.05, **Figure 2**).

### 3.4 Trends in clinical pregnancy and live birth rates across endometrial thickness in different ovulation induction protocols

**Figure 3:**
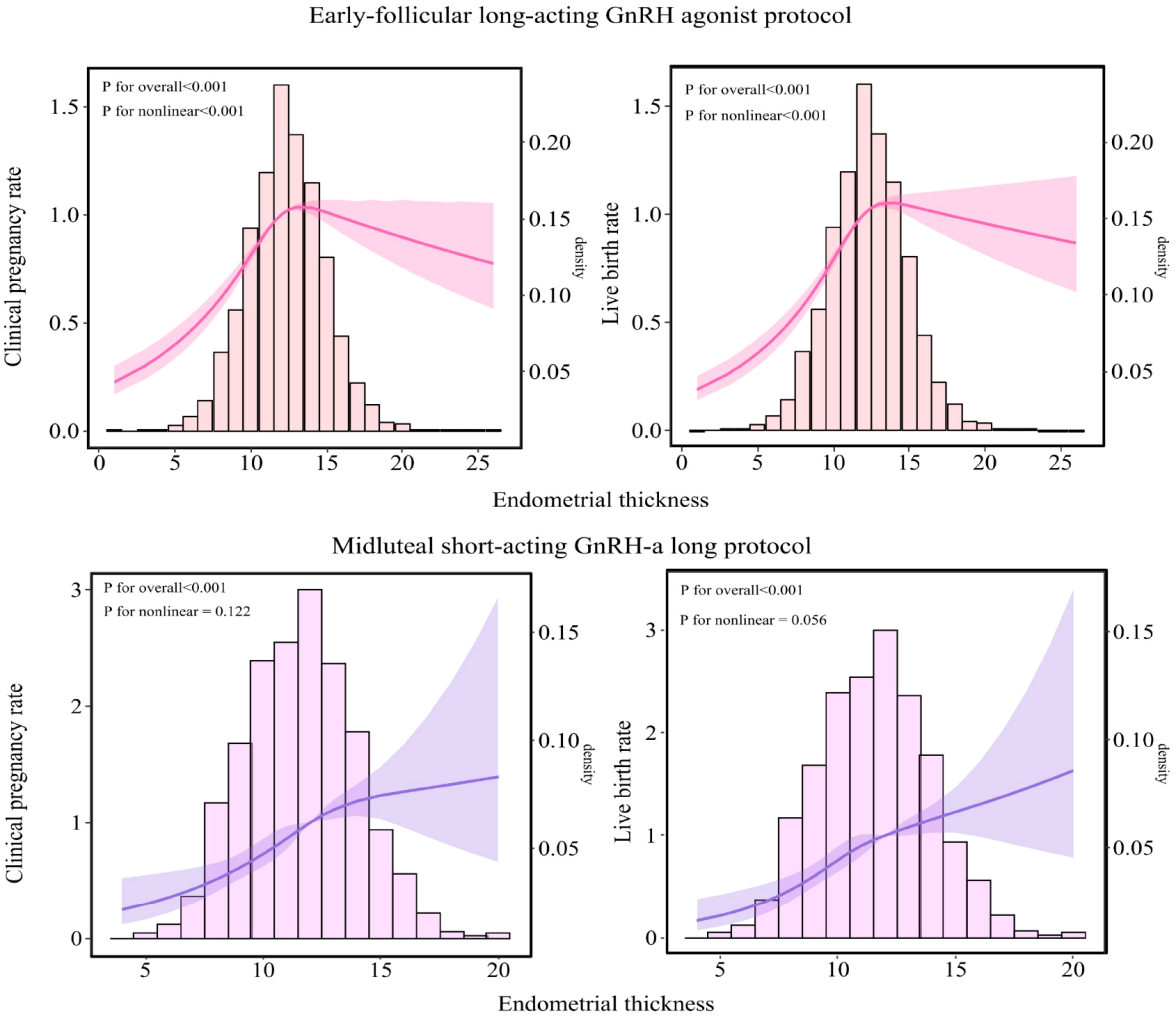
Relationship between endometrial thickness and pregnancy outcomes in populations undergoing different ovarian stimulation protocols. The RCS was used to calculate the estimated changes (95% CIs) adjusted for age, BMI, infertility type, number of embryos transferred, Gn initiation, total Gn, number of sinus follicles, number of follicle retrievals, and controlled ovulation induction protocol. CI, confidence interval; BMI, body mass index; Gn, gonadotropin.

As illustrated in **Figure 3**, among patients undergoing the early-follicular long-acting GnRH agonist protocol, endometrial thickness exhibited a nonlinear relationship with both clinical pregnancy and live birth rates (*P* for nonlinear < 0.05). In contrast, for those following the midluteal short-acting GnRH-a long protocol, a linear association was observed (*P* for nonlinear > 0.05).

Specifically, in the early-follicular long-acting protocol group, when endometrial thickness was less than 10.6 mm, increases in thickness were associated with significant improvements in clinical pregnancy and live birth rates (aOR = 1.239; 95% CI: 1.183–1.297 and aOR = 1.229; 95% CI: 1.181–1.279, respectively; **Table 3**). However, when endometrial thickness reached or exceeded 10.6 mm, these rates plateaued, showing no further significant changes (all P > 0.05).

**Table 3:** Segmented logistic regression analysis of endometrial thickness on clinical pregnancy and live birth rates across different ovulation induction protocols.

Segmented logistic regression	aOR(95%CI)	*P*
Follicular length program		
Clinical pregnancy rate		
Endometrial thickness <10.6	1.239(1.183-1.297)	<0.001
Endometrial thickness ≥10.6	1.018(1.002-1.034)	0.028
Live birth rate		
Endometrial thickness <10.6	1.229(1.181-1.279)	<0.001
Endometrial thickness ≥10.6	1.016(0.998-1.033)	0.066
Long luteal phase program		
Clinical pregnancy rate		
Endometrial thickness	1.128(1.087-1.170)	<0.001
Live birth rate		
Endometrial thickness	1.132(1.091-1.175)	<0.001

We performed multivariate logistic regression, adjusting for age, BMI, infertility type, number of embryos transferred, Gn initiation, total Gn, number of sinus follicles, number of follicle retrievals, and controlled ovulation induction protocol. CI=confidence interval, BMI=body mass index, Gn=gonadotropin; EFLL=early-follicular long-acting GnRH agonist protocol; MLSL= midluteal short-acting GnRH agonist long protocol.

Conversely, in the midluteal short-acting protocol group, both clinical pregnancy and live birth rates increased steadily with endometrial thickness across the entire range studied (aOR = 1.128; 95% CI: 1.087–1.170 and aOR = 1.132; 95% CI: 1.091–1.175, respectively; **Table 3**).

### 3.5 Trends in miscarriage and preterm birth rates across endometrial thickness in different ovulation induction protocols

**Figure 4:**
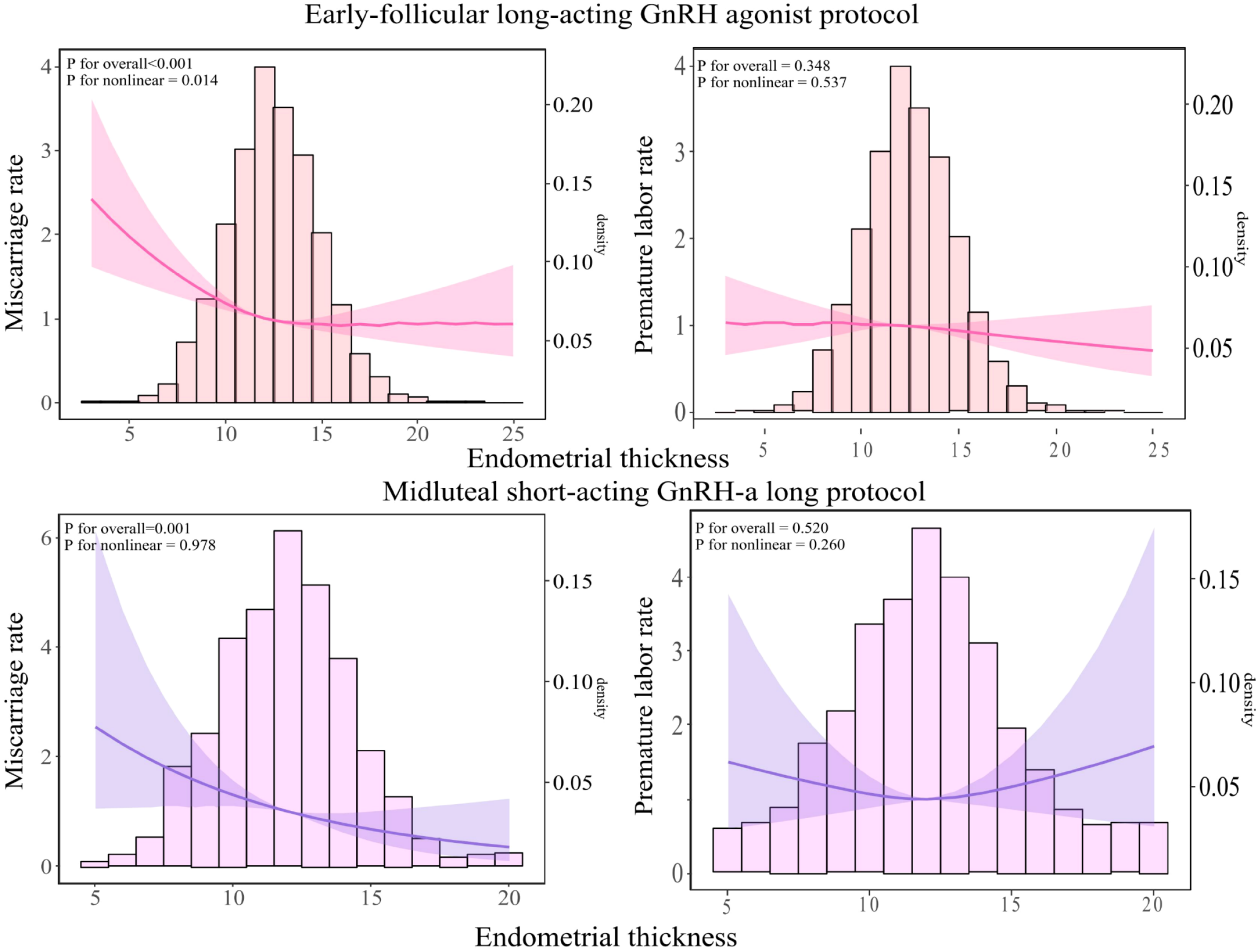
Relationship between endometrial thickness and miscarriage and live birth rates across different ovulation induction protocols. The RCS was used to calculate the estimated changes (95% CIs) adjusted for age, BMI, infertility type, number of embryos transferred, Gn initiation, total Gn, number of sinus follicles, number of follicle retrievals, and controlled ovulation induction protocol. CI, confidence interval; BMI, body mass index, Gn, gonadotropin.

As illustrated in **Figure 4**, among patients undergoing the early-follicular long-acting GnRH agonist protocol, endometrial thickness exhibited a nonlinear relationship with miscarriage rates (*P* for nonlinear < 0.05). In contrast, for those following the midluteal short-acting GnRH-a long protocol, a linear association was observed *(P* for nonlinear > 0.05).

Specifically, in the early-follicular long-acting protocol group, when endometrial thickness was less than 15 mm, increases in thickness were associated with a significant decrease in miscarriage rates (aOR = 0.93; 95% CI: 0.905–0.956; **Table 4**). However, when endometrial thickness reached or exceeded 15 mm, miscarriage rates plateaued, showing no further significant changes *(P* = 0.087).

**Table 4:** Segmented logistic regression analysis of endometrial thickness on miscarriage and preterm birth rates across different ovulation induction protocols.

Segmented logistic regression	aOR(95%CI)	*P*
Long follicular phase program		
Miscarriage rate		
Endometrial thickness <15	0.93(0.905-0.956)	<0.001
Endometrial thickness ≥15	1.073(0.988-1.16)	0.087
Preterm birth rate		
Endometrial thickness	0.985(0.964-1.008)	0.199
Long luteal phase program		
Miscarriage rate		
Endometrial thickness	0.88(0.816-0.941)	<0.001
Preterm labor rate		
Endometrial thickness	1.008(0.94-1.081)	0.822

We performed multivariate logistic regression, adjusting for age, BMI, infertility type, number of embryos transferred, Gn initiation, total Gn, number of sinus follicles, number of follicle retrievals, and controlled ovulation induction protocol. CI, confidence interval, BMI, body mass index, Gn, gonadotropin; EFLL, early-follicular long-acting GnRH agonist protocol; MLSL, midluteal short-acting GnRH agonist long protocol.

Conversely, in the midluteal short-acting protocol group, miscarriage rates decreased steadily with increasing endometrial thickness across the entire range studied (aOR = 0.88; 95% CI: 0.816–0.941; **Table 4**).

Notably, in both protocols, endometrial thickness was not significantly associated with preterm birth rates (all *P* > 0.05).

## 4 Discussion

Multiple studies have explored the relationship between endometrial thickness and pregnancy outcomes, as well as the optimal threshold (13–16). The association between endometrial thickness and pregnancy outcomes in assisted reproductive technology (ART) cycles is nonlinear. Large-scale retrospective studies have shown that as endometrial thickness increases from extremely thin to an optimal range (approximately 10–12 mm), clinical pregnancy rates and live birth rates improve significantly. For example, Xu et al., analyzing data from 42,132 fresh embryo transfer cycles, found that for every 1 mm increase in endometrial thickness below 12 mm, the clinical pregnancy rate (CPR) and live birth rate (LBR) increased by approximately 9–10% (16). However, when endometrial thickness reached 12–15 mm, the pregnancy rates tended to plateau. In contrast, when the endometrium is too thin (<7 mm), implantation success rates are significantly reduced due to insufficient glandular development, inadequate vascularization, and hormonal support, and this is also associated with a higher miscarriage rate (17). To date, no studies have compared the trends of endometrial thickness and pregnancy outcomes, as well as clinical pregnancy and perinatal outcomes at different endometrial thickness levels, between the early-follicular long-acting GnRH agonist protocol and the midluteal short-acting GnRH-a long protocol. Therefore, this study investigated the impact of changes in endometrial thickness on clinical outcomes under these two ovarian stimulation protocols. The results showed that in the population undergoing the early-follicular long-acting protocol, endometrial thickness exhibited a pattern of initially increasing and then plateauing in relation to clinical pregnancy rate and live birth rate, while miscarriage rate showed an initial decrease followed by stabilization. When endometrial thickness was less than 10.6 mm, pregnancy rates increased significantly with increasing thickness; however, beyond 10.6 mm, pregnancy rates no longer improved with further increases in endometrial thickness. This is consistent with the findings of Mahutte N et al. in 2022 and Gallos ID et al. in 2018 (6, 14). In contrast, among patients receiving the midluteal short-acting GnRH-a long protocol, endometrial thickness was positively correlated with clinical pregnancy rate and live birth rate, and negatively correlated with miscarriage rate. These findings are consistent with those reported by Zhang J et al (18).

There is still controversy regarding whether an excessively thick endometrium adversely affects pregnancy outcomes. Xu J et al. found that when endometrial thickness is ≥15 mm, both clinical pregnancy rate and live birth rate decrease (16). Most studies have shown that an excessively thick endometrium does not adversely affect pregnancy outcomes (13, 14, 19, 20). Recent meta-analyses indicate that a thick endometrium (>14 mm) overall does not reduce the chance of pregnancy (17), with only a thin endometrium significantly lowering pregnancy success rates. Therefore, the current consensus is that the optimal endometrial thickness (EMT) is approximately 8–12 mm; an extremely thin endometrium is clearly detrimental, while the effects of very high thickness remain inconclusive (17), which aligns with the findings of this study. Although some studies have suggested that an excessively thick endometrium may negatively affect pregnancy outcomes, our findings—together with existing evidence on endometrial blood perfusion and receptivity marker expression—indicate that endometrial thickening is not necessarily detrimental. Our results demonstrate that an endometrial thickness ≥15 mm does not negatively impact pregnancy outcomes. On the contrary, in the midluteal short-acting GnRH-a long protocol group, pregnancy rates continued to increase as endometrial thickness exceeded 15 mm.

Previous studies have shown that the expression levels of endometrial receptivity markers HOXA10, MEIS1, and LIF (21–25) are higher in patients undergoing the early-follicular long-acting GnRH agonist protocol compared with those receiving the midluteal short-acting long protocol (11), suggesting that the follicular-phase long protocol confers better endometrial receptivity (26). This indicates that long-acting GnRHa administration may improve the implantation environment by enhancing the expression of genes associated with endometrial receptivity, which could explain why the follicular-phase long protocol achieves a relatively high pregnancy rate even within the moderate endometrial thickness range.

On the other hand, the duration and dosage of GnRHa pretreatment can also affect the expression of endometrial cytokines. For instance, studies have reported that GnRHa treatment upregulates IL-11 and integrin αvβ3 expression in the endometrium, thereby promoting trophoblast invasion and supporting early pregnancy maintenance (27). In addition, adequate endometrial blood supply and vascular development are essential for maintaining normal endometrial function and receptivity. Endometrial proliferation and differentiation depend on sufficient blood flow to deliver oxygen, nutrients, and hormones while removing metabolic waste products. Vascular endothelial growth factor (VEGF) is abundantly expressed during the proliferative phase to stimulate microvascular dilation and neovascularization, ensuring rich perfusion during the implantation window. In contrast, excessively thin endometrium is often associated with poor perfusion and sparse vasculature, which can be reflected by an elevated resistance index (RI) of the uterine radial artery (RA-RI) on Doppler ultrasound (28). A study focusing on “thin” endometrium demonstrated that, compared with women with normal endometrial thickness, patients with thin endometrium showed significantly higher uterine radial artery resistance throughout the cycle, along with reduced glandular development, decreased vascular density, and markedly lower VEGF expression during the mid- to late-proliferative phases (28). These findings suggest that inadequate vascularization and limited blood flow in a thin endometrium lead to poor nutrient and oxygen supply, thereby compromising embryo survival and implantation potential. This also explains why endometrial thickness below 7 mm is often associated with low pregnancy rates.

Clinical intervention studies have further shown that improving uterine blood flow—through supplementation with vitamin E, L-arginine, or sildenafil citrate, among others—can reduce radial artery resistance and increase endometrial thickness (29). Enhanced local perfusion provides a more oxygenated and nutrient-rich endometrial microenvironment that better supports embryo implantation. When endometrial thickness reaches approximately 10–12 mm, an optimal range, the vascular bed is typically well established: spiral arteries have undergone remodeling, microvascular density is increased, and vascular resistance is reduced. At this stage, the endometrium receives adequate perfusion throughout all layers, creating a stable environment for embryo implantation and exchange of nutrients and gases. Further thickening beyond this point does not significantly improve blood flow, as perfusion is no longer the limiting factor.

Our study also showed that when endometrial thickness reaches or exceeds 15 mm, both protocols achieve similar pregnancy rates. This may be because increased endometrial thickness is associated with reduced resistance in uterine radial artery blood flow and improved vascular development (29, 30), thereby significantly enhancing endometrial receptivity and resulting in comparable pregnancy outcomes between the two protocols. Overall, the pregnancy rate advantage of the early-follicular long-acting protocol may be related to the upregulation of endometrial receptivity markers such as HOXA10 and LIF, while the midluteal short-acting protocol may achieve similar outcomes in cases of thicker endometrium by improving uterine blood flow.

Compared to the midluteal short-acting GnRH-a long protocol, the early-follicular long-acting GnRH agonist protocol is associated with a higher incidence of gestational diabetes mellitus (3.4% vs. 1.2%), which is consistent with previous findings by Du L and Wang D (31, 32). This may be related to the use of long-acting GnRH agonists potentially causing impaired glucose tolerance and increased insulin resistance (33). Additionally, studies have indicated that the dose of gonadotropins is a risk factor for the development of gestational diabetes mellitus (34), and the early-follicular long-acting protocol involves higher and longer gonadotropin usage during ovarian stimulation.

The relationship between endometrial thickness and preterm birth rate remains controversial. This study found no association between endometrial thickness and preterm birth rate in either protocol, which is consistent with several previously published studies (17, 35, 36). However, a 2023 study by Wu J et al. reported a negative correlation between endometrial thickness and preterm birth rate (37). This discrepancy may reflect differences in study design, sample selection, as well as regional and population variations. Furthermore, the mechanisms by which endometrial thickness might influence preterm birth remain unclear and may involve complex endocrine and uterine environmental factors. Therefore, prospective studies are needed in the future to clarify the relationship between endometrial thickness and preterm birth rate and to explore the underlying biological mechanisms.

This study is the first to use restricted cubic spline (RCS) analysis to investigate the differing trends between endometrial thickness and clinical pregnancy rates in IVF/ICSI patients undergoing the early-follicular long-acting GnRH agonist protocol and the midluteal short-acting GnRH-a long protocol. This study has several strengths. First, by using RCS to build nonlinear models, it provides a more precise characterization of how pregnancy rates vary with endometrial thickness. Second, the strict inclusion and exclusion criteria ensured that the study population closely represents the general patient population. Lastly, with a large sample size of 21,290 clinical records, the modeling results are more reliable. However, this study still has certain limitations. First, all cases were derived from a single reproductive medicine center. While this ensured consistency in treatment protocols and laboratory procedures, it may limit the external generalizability of the results. Second, as a retrospective study, it remains subject to potential selection and information biases, although we attempted to control measurable confounders through propensity score matching (PSM) and multivariate logistic regression. Nevertheless, due to limitations in the original clinical dataset, several potential confounding factors—such as lifestyle (diet, daily routine, psychological stress), environmental exposures, previous medication use, and hormonal imbalances—were not systematically collected and thus could not be incorporated into the model analysis. These unmeasured variables might have influenced the pregnancy outcomes to some extent.

In addition, because of the restricted study period and incomplete follow-up data, neonatal and long-term maternal outcomes were not available, making it difficult to assess the long-term health effects of different stimulation protocols. Future research should combine long-term follow-up cohorts to systematically evaluate the long-term safety and reproductive outcomes of various ovarian stimulation protocols. Moreover, prospective, multicenter studies with more comprehensive data collection are needed to further enhance the reliability and generalizability of the conclusions.

## Data Availability

The raw data supporting the conclusions of this article will be made available by the authors, without undue reservation.
